# Longer term effects of the Angelina Jolie effect: increased risk-reducing mastectomy rates in BRCA carriers and other high-risk women

**DOI:** 10.1186/s13058-015-0650-8

**Published:** 2015-11-25

**Authors:** D. Gareth Evans, Julie Wisely, Tara Clancy, Fiona Lalloo, Mary Wilson, Richard Johnson, Jonathon Duncan, Lester Barr, Ashu Gandhi, Anthony Howell

**Affiliations:** Genesis Breast Cancer Prevention Centre, University Hospital of South Manchester NHS Trust, Wythenshawe, Manchester, M23 9LT UK; Genomic Medicine, St. Mary’s Hospital, Manchester Academic Health Science Centre, Institute of Human Development, Central Manchester Foundation Trust, Oxford Road, Manchester, M13 9WL UK; Department of Psychology, University Hospital of South Manchester NHS Trust, Wythenshawe, Manchester M23 9LT UK; Nightingale Breast Screening Centre, University Hospital of South Manchester NHS Trust, Wythenshawe, Manchester M23 9LT UK; Department of Breast Surgery, University Hospital of South Manchester NHS Trust, Wythenshawe, Manchester M23 9LT UK; Department of Plastic Surgery, University Hospital of South Manchester NHS Trust, Wythenshawe, Manchester M23 9LT UK

In May 2013 the actress Angelina Jolie informed the press that she had undergone bilateral risk-reducing mastectomy (BRRM) because she carried a maternally inherited pathogenic *BRCA1* mutation. This decision created huge publicity worldwide [1] and led to enormous interest in hereditary breast cancer/genetic testing. Here we comment on our recently published research article in *Breast Cancer Research* and provide more recent observations. This reported a 2.5-fold increase in referrals of UK women with family histories of breast cancer 3–4 months following Ms Jolie’s revelation [1]. We also highlighted increased interest in BRRM; however, as it takes 9–12 months from initial BRRM enquiries to the operative procedure, we can now report a similar 2.5-fold increase in uptake of BRRM in the 6–24 months following this.

The Genesis Prevention Centre Family History clinic (GPCFHC) covers an extended population of around 5 million. Although the main impact of the Angelina effect was from June to November 2013, this trend continued through 2014 with increased referrals from 201 in January–June 2012 to 388 (odds ratio (OR) 1.93) in January to June 2014 and rising by 366 (OR 2.09) for the last 6 months to give a total of 754 for 2014. Women attending for risk assessment and discussions concerning BRRM, unprompted, still mention the effects of Angelina Jolie on their attendance anecdotally to clinic physicians and still reflect on the impact of her speaking publicly in their pre-surgery consultations with the clinical psychologist in 2015. A clear upward trend in BRRM can be seen starting around 6 months after the news announcement in May 2013 (Fig. 1). The number of high-risk women without *BRCA1/2* mutations undergoing BRRM (*n* = 12; 18 months from January 2011) rose to 52 (18 months from January 2014). The number in mutation carriers rose from 17 to 31. The overall combined rise from 29 BRRMs to 83 was significant (high-risk women at GPCFHC, *n* = 2012; chi-square *p* < 0.0001). Again BRRM numbers annually had been stable at around 20 (2000–2011). We speculate that the BRRM rate rise was probably contributed by the ‘Angelina effect’. This effect was seen not just in carriers of *BRCA1*/*BRCA2*, but was actually greater in those without mutations. Nonetheless, 23/31(74 %) BRRMs in mutation carriers were in women >18 months after testing positive, indicating a delay in decision-making, whereas prior to 2013 the majority of women had BRRM within 18 months of testing positive [2]. There was a slight rise in the number of unaffected women newly testing positive for *BRCA1/2* in Manchester from 81 to 116 in the 2 years before and the 2 years after Angelina’s announcement, although this could have been impacted by new National Institute for Health and Care Excellence (NICE) guidelines announced in June 2013 [3]. This research was exempt from ethical approval as this is an audit of clinical service and does not contain identifiable data.

**Fig. 1:**
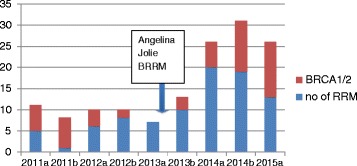
Number of BRRMs carried out at Wythenshawe and Christie hospitals per 6-month period from 2011 and proportion with mutations in high-risk genes. *a* January–June, *b* July–December, *red* proportion with BRCA1/2/TP53 mutations, *BRRM* bilateral risk-reducing mastectomy

The present audit of further new referrals and BRRM rates indicates that the Angelina effect has been prolonged and has impacted on increased referral and BRRM rates. It would be interesting to see results from centres worldwide. Plans to offer breast cancer risk assessment on a population basis could further affect uptake of BRRM [4]. It is also possible that similar effects will be seen on the already increasing rates of contralateral mastectomy in women with breast cancer [5].

## Abbreviations

BRRM: Bilateral risk-reducing mastectomy
GPCFHC: Genesis Prevention Centre Family History clinic
OR: Odds ratio
